# *ProLEED Studio*: software for modeling low-energy electron diffraction patterns

**DOI:** 10.1107/S1600576723010312

**Published:** 2024-02-01

**Authors:** Pavel Procházka, Jan Čechal

**Affiliations:** a CEITEC – Central European Institute of Technology, Brno University of Technology, Brno, Purkyňova 123, 61200, Czechia; b Institute of Physical Engineering, Brno University of Technology, Brno, Technická 2896/2, 61669, Czechia; Ecole National Supérieure des Mines, Saint-Etienne, France

**Keywords:** low-energy electron diffraction, LEED, *ProLEED* Studio, reciprocal space, computer programs

## Abstract

Low-energy electron diffraction patterns contain precise information about the structure of the surface studied. However, retrieving the real space lattice periodicity from complex diffraction patterns is challenging, especially when the modeled patterns originate from superlattices with large unit cells composed of several symmetry-equivalent domains without a simple relation to the substrate. This work presents *ProLEED Studio* software, built to provide simple, intuitive and precise modeling of low-energy electron diffraction patterns. The interactive graphical user interface allows real-time modeling of experimental diffraction patterns, change of depicted diffraction spot intensities, visualization of different diffraction domains, and manipulation of any lattice points or diffraction spots. The visualization of unit cells, lattice vectors, grids and scale bars as well as the possibility of exporting ready-to-publish models in bitmap and vector formats significantly simplifies the modeling process and publishing of results.

## Abstract 2

*ProLEED Studio* is software designed for simple, intuitive and precise modeling of low-energy electron diffraction patterns.

## 1. Introduction

Low-energy electron diffraction (LEED) is one of the oldest surface science techniques for indirect surface structure determination. It was discovered and demonstrated by Clinton Davisson and Lester Germer in 1927, who directed a beam of low-energy electrons onto a nickel crystal and observed its LEED pattern (Davisson & Germer, 1927*a* ▸,*b* ▸). The main LEED experimental expansion started in the 1960s with the rapid development of ultra-high vacuum systems. Since then, LEED has become the standard structure characterization technique (Jona *et al.*, 1982 ▸; Van Hove *et al.*, 1986 ▸; Moritz *et al.*, 2009 ▸; Moritz & Van Hove, 2022 ▸). In typical LEED experiments, electrons with an energy of 30–300 eV are focused and accelerated towards a clean crystalline surface, and elastically backscattered electrons are detected by a hemispherical fluorescent screen. The possibility of measuring LEED patterns is also an integral part of a low-energy electron microscope (Bauer, 1994 ▸, 2020 ▸; Tromp, 2000 ▸; Altman, 2010 ▸).

LEED patterns generally contain precise information about the surface structure: the positions of atoms in the topmost few layers. Their retrieval from the experimental data is a challenging task comprising modeling the structure within the dynamical theory of diffraction and comparing calculated spot intensities for a significant interval of primary electron energies. On the other hand, in most cases, the use of LEED is straightforward: a brief look at the diffraction pattern provides an experienced researcher with information on the surface periodicity and symmetry. If the diffraction patterns are relatively simple, the widely used *LEEDpat* software does excellent work in the visualization of diffraction patterns associated with real space lattices (Hermann & Van Hove, 2014 ▸). However, extracting the surface structure from complex experimental diffraction patterns is often non-trivial, especially when dealing with superlattice structures like those originating from molecular adsorbates (Procházka *et al.*, 2020 ▸, 2021 ▸; Makoveev *et al.*, 2022 ▸).

Here, we present the *ProLEED Studio* software, a tool designed for simple, precise and intuitive modeling of LEED patterns to obtain a 2D unit cell. It allows (i) loading of an experimental pattern and its direct comparison with the modeled diffraction pattern; (ii) drag and drop positioning of the lattice points both in real and in reciprocal space while observing instantaneous changes in the other space; (iii) changing the size of the modeled spots to simulate their distinct intensities, which is helpful if the complex patterns of molecular layers are analyzed; and (iv) pinning of the real space superstructure lattice point to the closest substrate lattice point, *i.e.* to obtain the closest commensurate lattice. Additionally, visualization of unit cells, vectors, superlattice domains and scale bars greatly simplifies the modeling. These functionalities also make *ProLEED Studio* an excellent educational tool. In the following text, we describe the theoretical background, functionality and graphical user interface (GUI) of *ProLEED Studio* and provide three examples of its application. We note that there are many other procedures that can be used for advanced evaluation of diffraction experiments [*e.g.* for description of glide planes, stepped surfaces, faceted surfaces, antiphase boundaries or modulated layers (Moritz & Van Hove, 2022 ▸)], but they are not included in the recent version of *ProLEED Studio*. Also, we do not include an intensity versus energy (I–V) curve analysis, which is targeted in recent efforts to make I–V analysis easy to use (*e.g.* by the *ViPErLEED* package; https://github.com/viperleed).

## 2. Theoretical background

In three dimensions, the diffraction condition is described by the Laue equation **k** − **k**_0_ = **G**, in which **k** and **k**_0_ are the wavevectors of the scattered and incident waves, respectively, and **G** is a vector of reciprocal space (Jona *et al.*, 1982 ▸; Van Hove *et al.*, 1986 ▸). At the surface, the bulk periodicity is truncated, and the Laue conditions are reduced to two equations regarding the components of the wavevectors parallel to the surface: 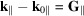 , where 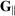 is a reciprocal lattice vector of the two-dimensional unit cell at the surface. In a LEED experiment, the incident wavevector **k**_0_ is typically perpendicular to the surface, and it is considered so in *ProLEED Studio*.

A two-dimensional solid surface is described by a 2D Bravais lattice 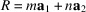 with the primitive basis vectors **a**_1_ and **a**_2_. A 2D reciprocal lattice in reciprocal space is associated with the Bravais lattice in real space. The primitive basis vectors 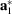 and 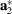 of the 2D reciprocal lattice are defined according to the orthogonality relation 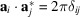 , with δ_*ij*_ being Kronecker delta and 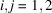 . With **n** being a unit vector perpendicular to the surface, the orthogonality relation gives the following expressions for reciprocal lattice vectors:

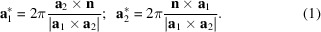

In a diffraction experiment, each diffracted beam corresponds to a reciprocal lattice vector 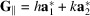 , where *h* and *k* are integers. Hence, a reciprocal lattice corresponds to the diffraction pattern with the exception of spot intensities.

We note that in crystallography the reciprocal lattice vector is often defined as **K** = **G**/2π and 2π is omitted from the equations given above, which simplifies specific mathematical manipulations. However, the majority of surface science textbooks (Van Hove *et al.*, 1986 ▸; Bechstedt, 2003 ▸; Lüth, 2015 ▸; Woodruff, 2016 ▸; Fauster, 2020 ▸) adhere to the definition used in solid-state physics, *i.e.* to include 2π in the definition of reciprocal vectors. We follow the definition in the field of surface science and include the factor 2π in the definition of reciprocal vectors in *ProLEED Studio*.

The superlattice with primitive basis vectors **s**_1_ and **s**_2_, 

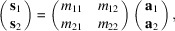

is considered in the same way as the substrate lattice; the associated primitive vectors of the reciprocal lattice are labeled 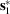 and 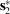 . The transformation matrix 

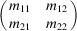

is used for the notation of superstructures in the matrix notation.

*ProLEED Studio* is based on the relation between real and reciprocal lattices: the diffraction pattern is represented as a reciprocal lattice. When changing or updating the real space lattice vectors **a**_1_ and **a**_2_, the reciprocal space lattice vectors are instantly recalculated and updated according to relations equivalent to equation (1):

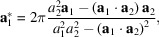




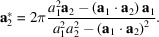

Similarly, the real space lattice vectors are updated using the relations

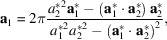




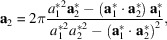

when a change of reciprocal space vectors is detected.

## 3. Graphical user interface

**Figure 1:**
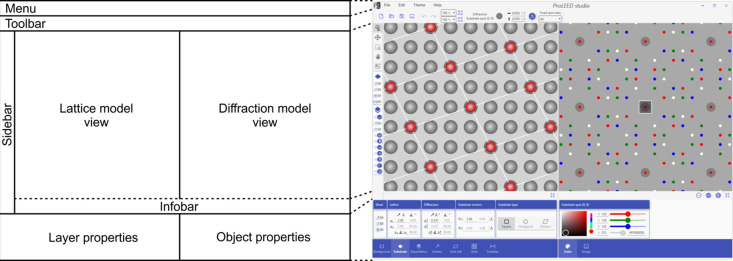
GUI of *ProLEED Studio* with a description of the main panels. The lattice and diffraction model view panels are used to visualize the lattice and diffraction models, respectively. Both allow direct interaction with and manipulation of selected objects. The menu, toolbar, sidebar, infobar, layer properties and object properties panels provide detailed information about the modeled system and allow the specification of all necessary properties.

Fig. 1 ▸ presents the control panel layout within the *ProLEED Studio* GUI. The main panels are the lattice model view and the diffraction model view. They serve for the visualization of the real and reciprocal lattice and direct drag-and-move manipulation with the lattice points. Both panels comprise different layers containing the background, grids, lattice points, diffraction spots, unit cells, arrows, the diffraction image and scale bars. The individual layers are described in more detail in the following section.

The menu panel on top of the *ProLEED Studio* GUI provides essential software functions for creating new models, saving or loading existing ones, importing diffraction images, and exporting models in vector or bitmap formats. It also allows the undo and redo actions for unit vector changes, provides the possibility of GUI color theme changes and contains a help menu. The toolbar below the menu panel provides shortcut buttons for menu functionalities and two boxes for updating the zoom level in the lattice and diffraction model views. Next, it contains detailed information about the selected object with the possibility to specify its properties.

The sidebar consists of three subpanels. The first subpanel allows the setting of different operational modes in lattice and diffraction model views, such as object selection, object movement, zooming, panning and diffraction image manipulation. The second and third subpanels are used to visualize objects related to the substrate and superlattice. Arrows, unit cells, grids, scale bars and different diffraction domains can be easily projected here.

The infobar below the lattice and diffraction model views shows the mouse cursor coordinates and hover object. It also contains buttons for the original view and quick changes between bright, dark and colored diffraction model view modes.

The layer properties panel is used for detailed parameter settings in individual layers of lattice and diffraction model views. The view sizes, number of lattice points, substrate and superlattice vectors, diffraction domains, and arrow, unit cell, grid and scalebar parameters can be read and set here.

The object properties panel briefly describes the selected objects and allows the setting of their color and transparency. More possibilities for selecting outline colors, thicknesses and 3D effects appear in the object properties panel when selecting specific objects such as lattice points, superlattice points and unit cells. Additional properties and information about the selected object appear in the toolbar menu. The object properties panel also contains a tab for diffraction image loading and manipulation.

## 4. Lattice and diffraction model layers

**Figure 2:**
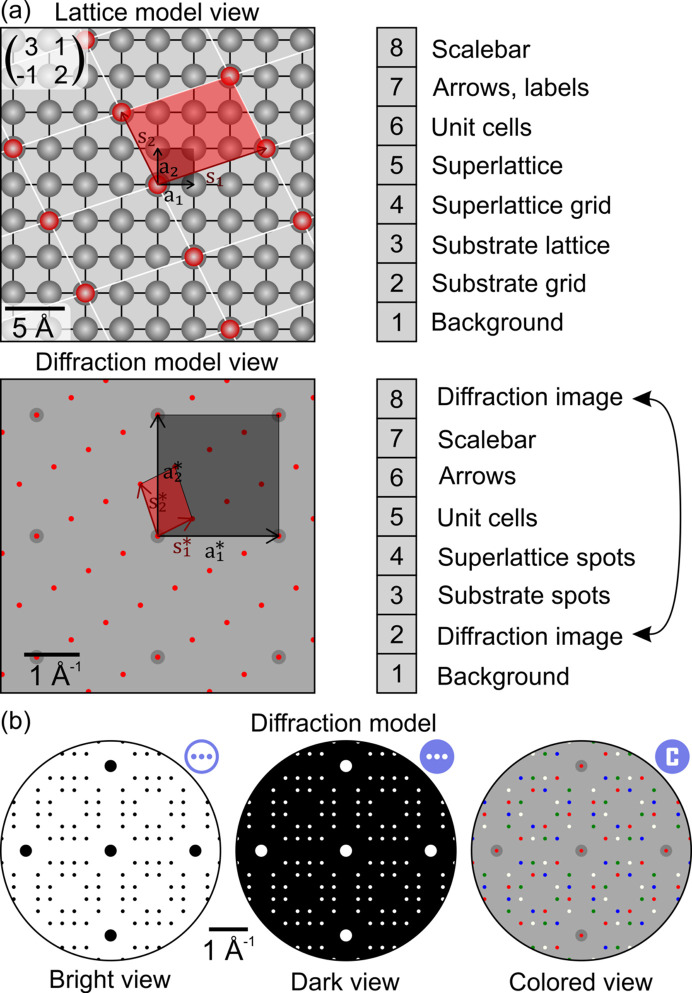
(*a*) *ProLEED Studio* lattice and diffraction model views consisting of different layers. Individual layers are plotted sequentially from the bottom (1) to the top (8). (*b*) Three distinct diffraction model view modes. The colored mode allows the color of spots to be changed within diffraction domains. The icons close to the diffraction model views represent icons in *ProLEED Studio* used for fast switching between different view modes.

The lattice and diffraction model views in *ProLEED Studio* comprise distinct layers for the background, grids, lattice points, diffraction spots, unit cells, arrows, the diffraction image and scale bars. The arrangement of the layers for both real space lattice and diffraction models is demonstrated in Fig. 2 ▸(*a*) for a superlattice expressed by 

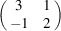

in matrix notation. The bottom-most layer, the background, is plotted first, and the other layers are rendered sequentially with increasing layer levels.

### 4.1. Background

The background is the bottom-most layer used to define the plotted area in real and reciprocal views. The size, color and transparency of the background can be set.

### 4.2. Grids

For convenient visualization of real space lattice points and unit cells, the lattice model view contains two additional layers for substrate and superlattice grids located directly underneath the substrate and superlattice point layers. Grids can be hidden, and their thickness and colors can be changed.

### 4.3. Lattice points and diffraction spots

Substrate and superlattice point layers in the lattice model view represent the periodical arrangement of lattice and superlattice points as a linear combination of the real space lattice vectors. Their number, colors, transparency, 3D effect, width and height, and rotation angle, as well as their outline color and thickness, can be changed.

Substrate and superlattice spot layers in the diffraction model view contain substrate and superlattice diffraction spots. The spot colors can be changed within the substrate or selected superlattice domain. The modeled spot sizes can be individually changed to simulate distinct experimental spot intensities.

### 4.4. Unit cells

In both the lattice and the diffraction model views, the unit-cell layers are located on top of the superlattice point layers. Their visualization can significantly simplify the interpretation and modeling of complicated structures. The unit-cell colors, transparencies and outline thicknesses can be set individually and projected for all substrate and superlattice domains.

### 4.5. Arrows and labels

Real and reciprocal space arrows that represent the lattice vectors for the substrate 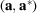 and superlattice 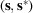 , together with their labels, can also be visualized. The arrow layers are located directly above the unit-cell layers. The arrow colors, thicknesses, label sizes and label colors can be set. The label positions are automatically updated when the lattice vectors are changed.

### 4.6. Scale bars

The scale bar layer is the topmost layer in the lattice and the second topmost layer in the diffraction model view. It allows the plotting and positioning of scale bars. The scalebar size, thickness, font size and color can be changed.

### 4.7. Diffraction image

The experimental diffraction images can be loaded into the topmost diffraction model view layer. However, for simple spot modeling, it can also be brought to the top of the background layer. The diffraction image size, rotation angle and transparency can be changed. The image can also be flipped horizontally or vertically.

## 5. Description of the *ProLEED* functionality

*ProLEED Studio* was built to provide simple, intuitive and precise modeling of experimental diffraction patterns. It allows real-time changes of modeled systems, undo–redo actions, superlattice domain visualization for different substrate types, the commensurate mode for superlattice models, zooming and panning of specific areas in both lattice and diffraction model views, and manipulation of the experimental diffraction patterns. Different intensities of diffraction spots can be visualized by changing the modeled spot sizes. The resulting models can be saved or exported in vector and bitmap formats.

The square, hexagonal or oblique substrate type can be selected when the initial model is created. If square or hexagonal types are selected, the substrate unit vectors are mutually coupled and updated together to preserve the selected substrate type. The lattice and diffraction unit vectors, their coordinates, the vector angles with the positive *x* axis, or the angles between vectors can be set in the layer properties tab. The substrate lattice arrangement can also be directly changed by dragging arbitrary points in the lattice or diffraction model views. Substrate diffraction spot sizes can all be changed at the same time, mirrored or individually.

*ProLEED Studio* allows the projection of the superlattice structure and the corresponding diffraction domains which can be independently visualized. Similarly to the substrate properties, superlattice parameters, including the matrix superlattice notation specification, can be set in the properties tab. *ProLEED Studio* also allows direct manipulation with first- and higher-order superlattice points in both real and reciprocal space. In this case, all the lattice vectors are updated automatically. For convenient diffraction pattern modeling, individual spot sizes can be changed.

The modeling and interaction with the superlattice can also be done in commensurate mode. In this mode, any of the selected real space superlattice points is, during its dragging, pinned to the closest substrate point, and the diffraction pattern is instantly updated.

## 6. Practical examples

The *ProLEED Studio* functionality is demonstrated in three practical examples. In all cases, we used an Ag(001) crystal as a substrate; its surface atoms are arranged in a square lattice with the interatomic distance *a* = 2.88 Å. In the first example, a simple substrate diffraction pattern is modeled, and the scalebar is added to the experimental result. The second and third examples illustrate the possibility of modeling more complicated superlattice arrangements (Procházka *et al.*, 2020 ▸; Makoveev *et al.*, 2022 ▸). In both cases, the crystal surface was covered by one monolayer (1 ML) of 4,4′-bi­phenyl­di­carb­oxy­lic acid (BDA) molecules with a distinct chemical state. As a result, the molecular layer in the second example forms a commensurate two-domain superlattice configuration with one molecule within the unit cell, and in the third example, a commensurate four-domain configuration with 42 molecules within the unit cell. Considering the size of the superlattice unit cell in the third example, we refer to it as the Moiré pattern. The typical experimental superlattice domain size was 800 × 800 nm in both cases. The detailed modeling process of all three practical examples is summarized in a video tutorial (http://y2u.be/SwCgiB2a7CQ).

### 6.1. Simple substrate

**Figure 3:**
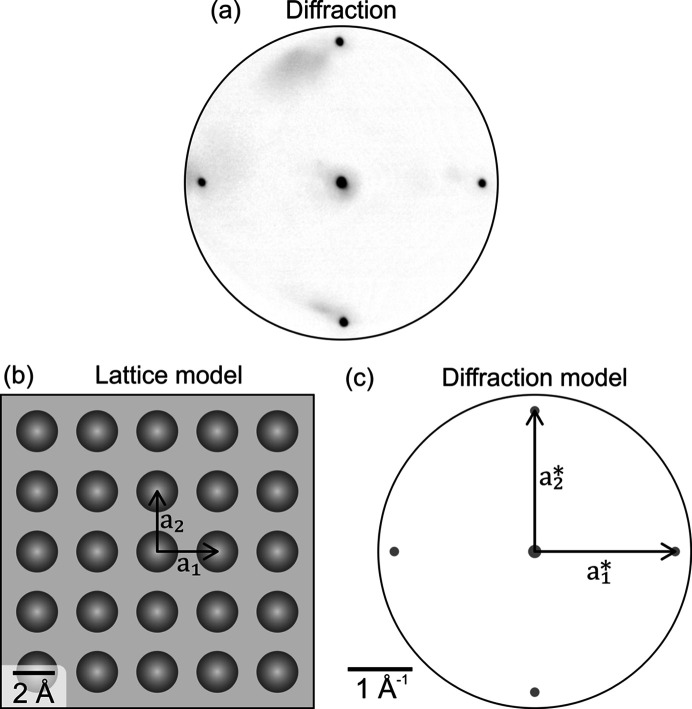
(*a*) Experimental diffraction pattern of an Ag(001) crystal measured by LEEM. The primary electron energy was 22 eV. (*b*) Lattice and (*c*) diffraction models simulated in *ProLEED Studio* using the substrate lattice constant *a* = 2.88 Å. Primitive vectors and scale bars are added in both real and reciprocal models.

The first example demonstrates the usage of *ProLEED Studio* for modeling the simple Ag(001) substrate and implementing the scalebar into an experimental diffraction pattern, shown in Fig. 3 ▸(*a*). The pattern was measured by LEEM using primary electrons with an energy of 22 eV. The four diffraction spots around the central one originate from substrate interatomic periodicity. The lattice and diffraction models in Figs. 3 ▸(*b*) and 3 ▸(*c*) were simulated using the known lattice parameter *a* = 2.88 Å. After the experimental dif­fraction image [Fig. 3 ▸(*a*)] had been loaded into *ProLEED Studio*, its image size was changed to overlap the substrate spots with the modeled ones to reach the same scale in both the model and the experiment. The resulting diffraction images can be easily exported with the correct scalebar.

### 6.2. Commensurate superlattice

**Figure 4:**
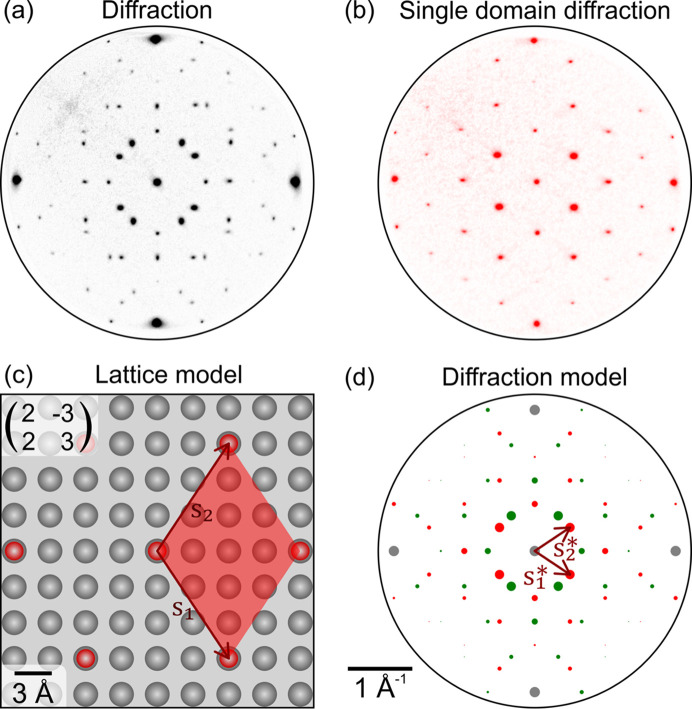
(*a*) Experimental diffraction pattern of the Ag(001) substrate covered by 1 ML of BDA molecules arranged in a two-domain superlattice configuration. (*b*) Single domain diffraction used for initial modeling. The pattern shows spots that belong to one superlattice domain. (*c*) Real space unit cell obtained by modeling a single domain diffraction pattern and pinning the superlattice points to the closest substrate ones. (*d*) Resulting diffraction model created by adjusting the spot sizes and projecting the second domain spots.

The diffraction pattern in Fig. 4 ▸(*a*) was measured on an Ag(001) substrate covered by 1 ML of BDA molecules arranged in a two-domain superlattice configuration with one molecule within the unit cell. The four most intense spots on the edges belong to the Ag substrate, whereas the rest of the diffraction spots belong to the superlattice. The superlattice diffraction pattern is composed of two identical subpatterns mutually rotated by 90°, which reflects the existence of two symmetry-equivalent superlattice domains mutually rotated by 90°, due to the substrate and superlattice symmetry (Makoveev *et al.*, 2022 ▸). The single domain diffraction in Fig. 4 ▸(*b*) was measured on an individual superlattice domain using a microdiffraction aperture, and was used for establishing the initial model. The real space model in Fig. 4 ▸(*c*) was obtained by changing the positions of modeled diffraction spots according to the measured data and pinning the superlattice points to the closest substrate points. The resulting superlattice can be expressed as 

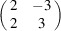

in matrix notation.

### 6.3. Moiré pattern

**Figure 5:**
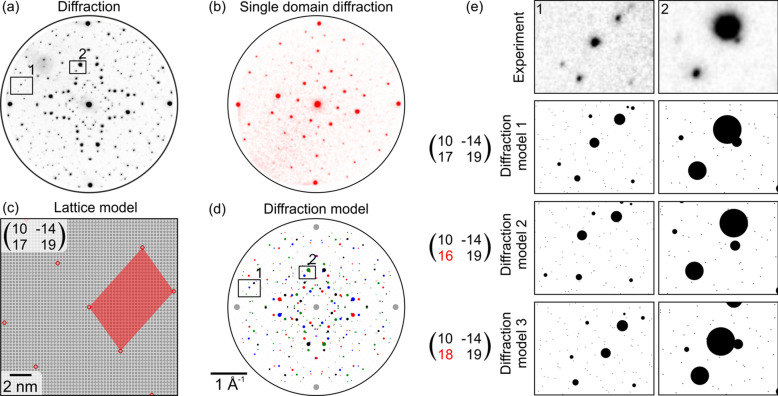
(*a*) Diffraction pattern of the Ag(001) substrate covered by 1 ML of BDA arranged in a four-domain structure. The pattern consists of four intense substrate spots around the edges and many Moiré spots originating from the long-range order superlattice periodicities. (*b*) Single domain diffraction pattern measured on an individual superlattice domain. (*c*) Real and (*d*) reciprocal models of Moiré periodicity. The diffraction pattern consists of two rotational and two mirror domains. (*e*) Illustration of the sensitivity of diffraction spot positions to small changes in superlattice models. Diffraction model 1 matches the experimental data, whereas slight changes in models 2 and 3 lead to apparent changes in spot positions that can be used for precise modeling.

Fig. 5 ▸(*a*) shows a diffraction pattern measured on an Ag(001) substrate covered by 1 ML of BDA molecules arranged in a four-domain structure commensurate with the substrate with a large unit cell (Procházka *et al.*, 2020 ▸). The initial diffraction model was obtained using the single domain diffraction pattern given in Fig. 5 ▸(*b*). By changing the superlattice spot positions, adjusting their sizes and pinning the superlattice point to the substrate, the resulting superlattice expressed by 

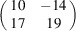

in the matrix notation was obtained. The superlattice model is depicted in Fig. 5 ▸(*c*). The diffraction model in Fig. 5 ▸(*d*) was subsequently created by the projection of all four diffraction domains.

The processing of the diffraction pattern measured by LEEM can be challenging as the diffraction patterns can be deformed by the magnetic lens projection system, rendering measurement of distances and angles in reciprocal space inaccurate. However, locally, the mutual position of diffraction spots from distinct domains presents a precise means for determining the structure. In this case, we seek local agreement (congruence) between the modeled and measured diffraction patterns. This is illustrated in Fig. 5 ▸(*e*), where two areas from Figs. 5 ▸(*a*) and 5 ▸(*d*) are enlarged, showing the spot positions for three close but distinct superlattice configurations. Diffraction model 1 shows a very good match with the experimental data. However, two slightly modified models, 2 and 3, show apparent differences in spot positions compared with the experiment. *ProLEED Studio* allows the positioning of zoomed-in higher-order diffraction spots, which allows tuning the mutual position of diffraction spots and, thus, simplifying the modeling process.

## 7. Software availability

The *ProLEED Studio* software is free and runs on a Windows platform. Its installer and experimental diffraction data shown in this article can be downloaded from its official website (https://proleedstudio.ceitec.vutbr.cz/). The software is written in C# (.NET Framework 4.7.2) and uses the Windows Presentation Foundation (WPF) graphical subsystem. The complete video tutorial can be found at http://y2u.be/SwCgiB2a7CQ.

## 8. Conclusions

In this paper, we present *ProLEED Studio* for simple, precise and intuitive modeling of experimental LEED patterns. The interactive GUI allows real-time manipulation with any lattice points or diffraction spots and projection of experimental patterns, superlattice domains, unit cells, vectors, grids and scale bars, significantly simplifying the modeling process. *ProLEED Studio* is also built for simple model importing or exporting of ready-to-publish images in bitmap or vector formats and includes undo and redo actions for unit vector changes. Its functionality was demonstrated through practical examples, including modeling a simple Ag(001) substrate, commensurate superlattice and complex Moiré pattern. *ProLEED Studio* holds great potential in accelerating the interpretation and understanding of surface structures and can significantly contribute to the advancement of surface science. Because of its simplicity, it can be employed as an educational tool.
